# Transcriptomic Analysis of Macrophage Polarization Protocols: Vitamin D_3_ or IL-4 and IL-13 Do Not Polarize THP-1 Monocytes into Reliable M2 Macrophages

**DOI:** 10.3390/biomedicines11020608

**Published:** 2023-02-17

**Authors:** Maria Rynikova, Petra Adamkova, Petra Hradicka, Jana Stofilova, Denisa Harvanova, Jana Matejova, Vlasta Demeckova

**Affiliations:** 1 Department of Animal Physiology, Faculty of Science, Pavol Jozef Safarik University in Kosice, 041 54 Kosice, Slovakia; 2 Faculty of Medicine, Institute of Clinical Medicine, University of Oslo, 0318 Oslo, Norway; 3 Center of Clinical and Preclinical Research MEDIPARK, Faculty of Medicine, Pavol Jozef Safarik University in Kosice, 040 11 Kosice, Slovakia; 4 Associated Tissue Bank, Faculty of Medicine, Pavol Jozef Safarik University in Kosice, 040 11 Kosice, Slovakia

**Keywords:** M1 macrophages, M2 macrophages, polarization protocol, transcriptomic analysis, differential gene expression, RNA-seq

## Abstract

Two main types of macrophages (Mφ) include inflammatory (M1) and anti-inflammatory (M2) macrophages. These cells can be obtained in vitro by polarization of monocytic cell lines using various stimuli. Since there is currently no consensus on the best method for the acquisition of reliable M1 and M2 macrophages from the THP-1 cell line, we decided to compare three different polarization protocols at the transcriptomic level. Whole transcriptomes of Mφ polarized according to the chosen protocols were analyzed using RNA-seq. Differential expression of genes and functional enrichment for gene ontology terms were assessed. Compared with other protocols, M1 macrophages polarized using PMA (61.3 ng/mL) and IFN-γ along with LPS had the highest expression of M1-associated regulatory genes and genes for M1 cytokines and chemokines. According to the GO enrichment analysis, genes involved in defensive and inflammatory processes were differentially expressed in these Mφ. However, all three chosen protocols which use Vit D_3_, IL-13/IL-4, and IL-4, respectively, failed to promote the polarization of macrophages with a reliable M2 phenotype. Therefore, optimization or development of a new M2 polarization protocol is needed to achieve macrophages with a reliable anti-inflammatory phenotype.

## 1. Introduction

Macrophages (Mφ) are part of the “monocyte-phagocyte system” which consists of tissue macrophages and their progenitors—monocytes present in the peripheral blood [1]. Recruited circulating monocytes leave the blood stream and differentiate into macrophages as a response to different stimuli in their respective tissue microenvironment [2]. Once differentiated, macrophages become long-lived cells and develop specialized functions. Cell numbers in the tissues are maintained by resistance to constitutive apoptosis, recruitment of further monocytes from the blood, and/or replication of local intermediates [3,4,5].

Mφ are essential for clearance of infections, induction of adaptive immunity, removal of old and dead cells and debris, promoting tissue repair, and wound healing [2,6]. Macrophages exercise all these functions via multiple key processes [2]:

- Phagocytosis (pathogens, debris, and dead and infected cells);
- Antigen presentation via MHCII molecules (major histocompatibility complex);
- Production of cytokines, chemokines, and growth factors.

For many years, the prevailing notion was that macrophages solely arose from the differentiation of circulating monocytes. However, more recent studies provided evidence that most adult tissue-resident macrophages are seeded before birth, derived from the yolk sac during embryonic development, and have a self-renewal capacity [7]. Tissue-resident macrophages play fundamental roles in tissue homeostasis and immune-surveillance. After the initial recognition of a microbial challenge, resident macrophages drive the influx of leukocytes, including monocytes, as a source of inflammatory macrophages. Therefore, the main role of monocyte-derived macrophages is host defense and subsequential post-inflammatory wound-healing and tissue remodeling [8].

Besides the basic distinction of tissue-resident and monocyte-derived macrophages, these cells can be divided into several sub-populations based on the differentiation stimuli and function [2]. In tissues, tissue-resident Mφ and monocytes recruited from the peripheral blood respond to different micro-environmental stimuli (e.g., damaged cells or pathogens and their products) with the acquirement of a functional phenotype [9]. Therefore, several classes of macrophages have already been described based mainly on the expression of surface markers, production of cytokines, chemokines, and growth factors, and their biological activities [10]. Two major and best characterized macrophage sub-populations are classically activated (M1) macrophages with their main role in host defense against pathogens, and alternatively activated (M2) macrophages (resolution of inflammation and tissue repair). This phenomenon of the two different M1/M2 phenotypes is referred to as “macrophage polarization”. However, it should be noted that M1 and M2 activation phenotypes represent two ends of a functional spectrum of macrophage polarization states [2,11]. On the other hand, some authors claim that due to the plasticity of Mφ, they constantly adapt to different micro-environmental stimuli. Since any analysis is only a “snapshot” of the current situation in the tissue, the plasticity of Mφ and their constant adaptations create an impression that there are numerous subtypes of macrophages [6,12].

Scientific papers use various nomenclature for the two main types of Mφ. Mostly they are referred to as M1/M2 macrophages or classically/alternatively activated macrophages. Another commonly used nomenclature is inflammatory (M1) and anti-inflammatory (M2) macrophages [11]. Although these terms are often used interchangeably, historically they represented macrophages that were identified under strikingly different conditions (reviewed in [6]). Some authors have proven that based on the gene expression, M1/M2 terms should not be considered equivalent to classically/alternatively activated Mφ [13]. In this article, we will be referring to polarized Mφ mainly as M1/M2 for simplicity and due to the common usage of this nomenclature across the scientific community. Still, it is important to acknowledge that according to the historical aspects of the nomenclature only, Mφ in the presented study should be considered rather as classically/alternatively activated.

There are several ways to study the differentiation and function of macrophages in vitro. The most accurate way to study tissue macrophages is to obtain specific Mφ from the tissue of interest. However, the isolation of these cells requires blood donation or invasive procedures, such as tissue biopsy, which often yield low amounts of tissue samples [14,15]. Moreover, Mφ isolated from the tissues have only a limited capacity to proliferate therefore only a relatively small number of cells is available for analysis [16,17]. To overcome this problem, immortalized monocytic cell lines of varying degrees of differentiation (e.g., KG1, HL-60, U937, and THP-1) are frequently used to model macrophage differentiation and function [18]. One of the most used monocytic cell lines is THP-1 (Tohoku Hospital Pediatrics-1 cells), which displays commitment towards macrophage differentiation. This cell line was isolated from a boy with acute monocytic leukemia by Tsuchiya et al. [19]. It resembles many aspects of human monocytes, such as morphology, expression of membrane antigens, and secretory products [18]. While differentiation of macrophages using monocytic cell lines such as THP-1 has obvious advantages in terms of simplicity, cost effectiveness, and ease of acquisition, results from these experiments may not always accurately predict the behavior of differentiated tissue macrophages [5,17]. To address this, differentiation protocols have been developed to obtain macrophages, which best resemble differentiated tissue macrophages regarding their function as well as their morphology, surface markers, gene expression, etc. [17]. Most of the protocols for polarization of M1 and M2 macrophages use cytokine stimuli [5]. First, the monocytes differentiate into “M0” macrophages, which represent a transitory state. This so-called activation of macrophages is obtained using phorbol-12-myristate-13-acetate (PMA). Activation is followed by polarization into M1 macrophages using interferon gamma (IFN-γ) and a toll-like receptor (TLR) agonist, such as lipopolysaccharides (LPS). Alternatively, M2 macrophages are polarized by stimulation with interleukin 4 (IL-4) and IL-13 [20,21]. However, some protocols use phorbol-12-myristate-13-acetate (PMA) as the sole polarization stimulus for M1 macrophages and 1,25-(OH)_2_-Vitamin D_3_ (Vit D_3_) to generate M2 macrophages [17,22].

The initial steps of biological research often depend on using cell cultures and in vitro assays to predict the function of different cells, their physiology, pathological changes, and treatment options in vivo. Therefore, besides the selection of the most appropriate and representative cell line, the most important component of in vitro experiments is the protocol used to stimulate and/or differentiate said cells. A standardized protocol is crucial to ensure the reproducibility of the experiment and most importantly the conditions which resemble the in vivo environment the most. The development of such a protocol is necessary to obtain results that would be relevant and applicable in in vivo settings. However, even though macrophages differentiated from the THP-1 cell line are widely used to study the function of human macrophages, there is currently no consensus on the best protocol for macrophage polarization from THP-1 cells [21,23]. Therefore, we decided to compare different polarization protocols from the perspective of the transcriptome of M1/M2 macrophages polarized according to these protocols. Firstly, we chose a protocol that uses PMA and Vit D_3_, and a protocol that involves activation with PMA followed by stimulation with cytokines. Lastly, we involved a third protocol in our analysis which also uses cytokine stimuli but applies a markedly different concentration of PMA to obtain M0 macrophages and a rest period between the activation phase and polarization of macrophages. The authors of this protocol claim that high concentrations of PMA block the subsequent polarization of the M2 macrophages [21].

## 2. Materials and Methods

### 2.1. THP-1 Cell Culture and Macrophage Polarization

**Figure 1:**
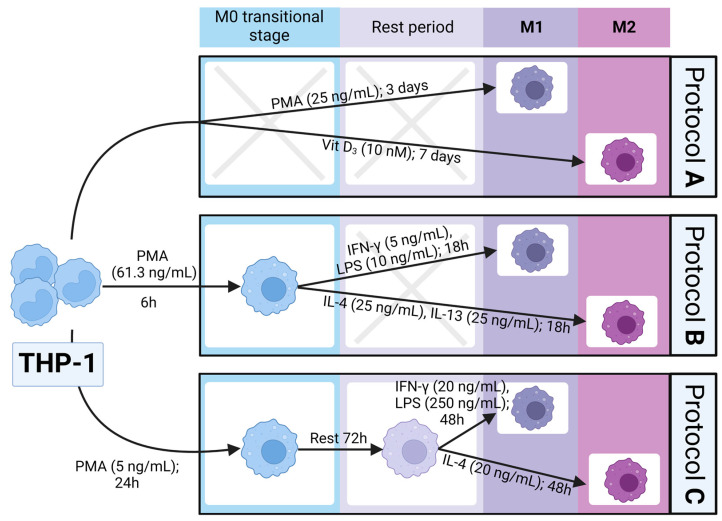
Different polarization protocols of M1 and M2 macrophages. THP-1—(Tohoku Hospital Pediatrics-1 cells) human monocytic cell line; PMA—phorbol 12-myristate 13-acetate; Vit D_3_—1,25-(OH)_2_-Vitamin D_3_; IFN-γ—interferon gamma; LPS—lipopolysaccharides; IL—interleukin. Created with BioRender.com; accessed on 15 December 2022.

THP-1 cells (ECACC, Salisbury, U.K.) were cultured at 37 °C with 5% CO_2_ in RPMI 1640 medium with stable L-glutamine supplemented with 10% (*v*/*v*) fetal bovine serum which were both obtained from Biosera (Nuaille, France). THP-1 monocytes were subsequently differentiated and polarized into M1 (classically activated) and M2 (alternatively activated) macrophages according to three different protocols (Figure 1):

Protocol A [22]: THP-1 cells were cultured in the presence of PMA (25 ng/mL, Sigma-Aldrich, St. Louis, MO, USA) for 3 days or Vit D_3_ (10 nM, Sigma-Aldrich, St. Louis, MO, USA) for 7 days to generate M1-like and M2 phenotypes, respectively.

Protocol B [20]: THP-1 cells were first cultured in the presence of PMA (61.3 ng/mL, Sigma-Aldrich, St. Louis, MO, USA) for 6 h to obtain an M0 phenotype and subsequently they were incubated for 18 h with either IFN-γ (5 ng/mL, Sigma-Aldrich, St. Louis, MO, USA) and lipopolysaccharides (LPS, 10 ng/mL, Sigma-Aldrich, St. Louis, MO, USA) or IL-4 (25 ng/mL, Peprotech, Cranbury, NJ, USA) and IL-13 (25 ng/mL, Stemcell Technologies, Vancouver, BC, Canada) to polarize into M1 and M2 macrophages, respectively.

Protocol C [21]: THP-1 cells were first stimulated with PMA (5 ng/mL, Sigma-Aldrich, St. Louis, MO, USA) for 24 h, then washed and allowed to rest in a fresh medium for 72 h. After the rest period, the primed M0 macrophages were stimulated for 48 h either with IFN-γ (20 ng/mL, Sigma-Aldrich, St. Louis, MO, USA) and LPS (250 ng/mL, Sigma-Aldrich, St. Louis, MO, USA) or IL-4 (20 ng/mL, Peprotech, Cranbury, NJ, USA) for M1 and M2 polarization, respectively.

Each macrophage subset was generated in three biological replicates in two independent experiments.

### 2.2. Live Cell Visualization of Macrophage Subtypes

For the purposes of morphological analysis, polarized macrophage subsets were visualized using the IncuCyte^®^ ZOOM reader (Essen Bioscience, Ann Arbor, MI, USA). Prior to adding polarization stimuli, THP-1 monocytes were seeded in 96-well plates (Sarstedt, Nümbrecht, Germany) with a seeding density of 1 × 10^4^ cells per well. After the polarization of each macrophage subset, the plate was placed in an incubator with the IncuCyte^®^ ZOOM reader (Essen Bioscience, Ann Arbor, MI, USA). The plate was imaged every 30 min for 24 h with 10× objective using the brightfield channel. The images were analyzed using the IncuCyte^®^ Zoom software (version 2015A; Essen Bioscience, Ann Arbor, MI, USA) and Image J [24] and subsequently assembled into figures in an online application BioRender (BioRender, Toronto, ON, Canada).

### 2.3. RNA Isolation

After the polarization of macrophage subsets, the cells were washed with fresh growth medium once, followed by washing with phosphate-buffered saline (PBS, Santa Cruz Biotechnology, Dallas, TX, USA). The RNA extraction was then performed according to an optimized protocol [25] with three chloroform extractions instead of two. As a phenol-based reagent for the initial RNA extraction, we used TRI Reagent^®^ (molecular Research Centre, Cincinnati, OH, USA). RNA was isolated from M0 macrophages from Protocol B and Protocol C, and from M1 and M2 macrophages from all three protocols. The concentration and purity of the extracted RNA were assessed using NanoDrop^TM^ One^C^ UV-Vis Spectrophotometer (Thermo Fisher Scientific, Waltham, MA, USA), and RNA integrity was evaluated by electrophoresis using 1.5% agarose gel. All samples were stored at −20 °C until library preparation.

### 2.4. Transcriptome Analysis

RNA library preparation, sequencing, and statistical analysis of the RNA-seq data were conducted by Novogene Europe (Cambridge, UK). Alignment to the reference genome was performed using HISAT2 [26]. Differential gene expression analysis was performed using edgeR software [27] with the Benjamini–Hochberg method as a false discovery rate (FDR) controlling procedure. The differentially expressed gene screening threshold was set as |log_2_ (FoldChange)| ≥ 1 with an adjusted *p*-value of padj ≤ 0.05. GO (gene ontology) enrichment analysis was performed using the clusterProfiler software [28].

## 3. Results

### 3.1. Visualization and Morphological Analysis

**Figure 2:**
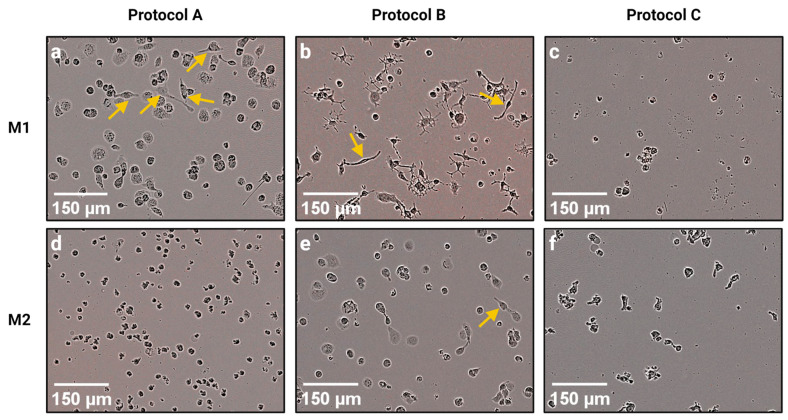
Visualization of polarized macrophages. Images were obtained immediately after removing the polarization stimuli for each Mφ subset (for time points see Figure 1) by the IncuCyte^®^ ZOOM reader with 10× objective using the brightfield channel. Yellow arrows point to cells with spindle-like morphology. (**a**) M1_A; (**b**) M1_B; (**c**) M1_C; (**d**) M2_A; (**e**) M2_B; (**f**) M2_C. Created with BioRender.com; accessed on 12 December 2022.

We identified great differences in cell numbers and morphology between M1 and M2 macrophage subtypes but also between different polarization protocols (Figure 2). After the polarization, we observed far less cells in the wells containing M1_C and M2_C (Figure 2c,f). Most of M1_A macrophages and all M2_A macrophages had a round shape without ruffles or filopodia (Figure 2a,d). Some of the M1_A cells had an elongated spindle-like morphology (Figure 2a, yellow arrows). The macrophages were first visualized immediately after the polarization. However, we observed that the morphology of cells was stable and did not change in the next 24 h during which the cells were monitored.

**Figure 3:**
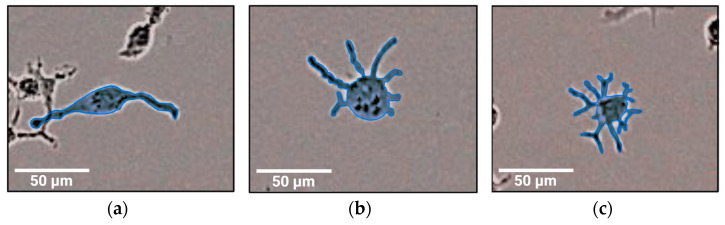
Morphological analysis of M1_B macrophages. Images were obtained immediately after removing the polarization stimuli for each Mφ subset (for time points see Figure 1) by the IncuCyte^®^ ZOOM reader with 10× objective using the brightfield channel. Morphology of the cell was then outlined and highlighted in BioRender (BioRender, Toronto, ON, Canada). (**a**) Macrophage with spindle-like morphology; (**b**) macrophage with filopodia; (**c**) macrophage with numerous ramified filopodia. Created with BioRender.com; accessed on 7 December 2022.

We observed several different shapes of M1 macrophages polarized according to Protocol B (Figure 2b and Figure 3). A few cells were of a rounded shape. Some M1 macrophages had an elongated spindle-like morphology, similar to M1_A (Figure 2a,b, yellow arrows; Figure 3a). Most of the M1_B macrophages had a morphology resembling a dendritic cell (DC) with several projections. Some of these M1 macrophages with a DC-like morphology had a few simple projections (Figure 3b) while others possessed numerous ramified filopodia (Figure 3c). The great majority of the M2_B macrophages had a rounded shape. However, occasionally we observed a cell with a spindle-like morphology (Figure 2e, yellow arrows).

M1_C and M2_C macrophages had overall a rounded, ragged, or slightly elongated shape (Figure 2c,f).

### 3.2. Global Analysis of Transcriptome Profiles

**Figure 4:**
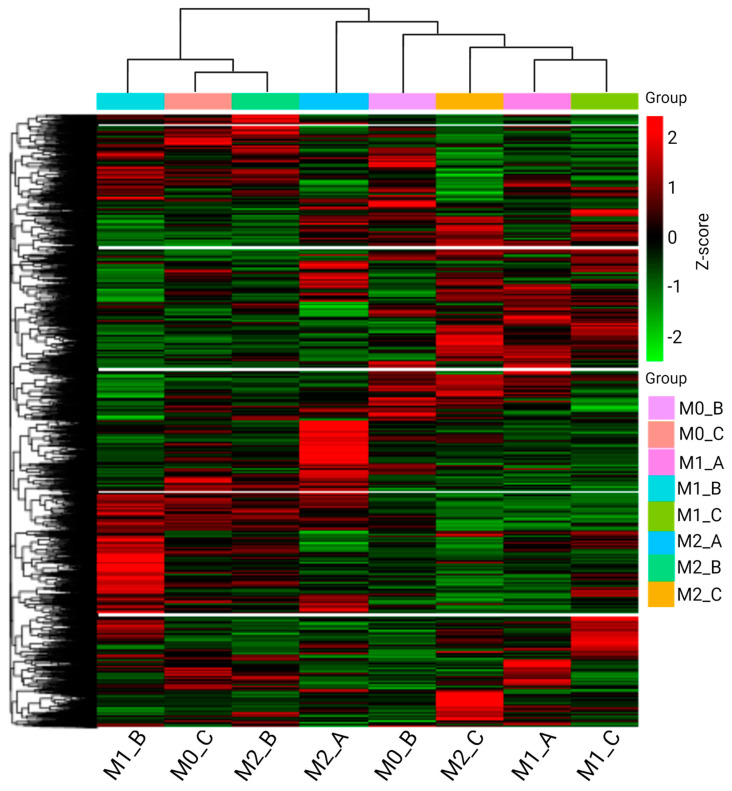
Hierarchical clustering (HCL). HCL analysis of transcriptome profiles of M0, M1, and M2 macrophages from all three chosen polarization protocols. Mainstream hierarchical clustering was performed using the Log2 FPKM+1 values of genes. Expression data rows were then homogenized to obtain the Z-score. Mφ subtypes of different protocols are listed in the x axis and genes are reported in the y axis. FPKM—fragments per kilobase of transcript sequence per million base pairs sequenced. Created with BioRender.com; accessed on 14 November 2022.

Firstly, hierarchical clustering (HCL) was performed to compare the transcriptomic profiles among different polarization protocols and among different macrophage polarization subtypes. The results of the HCL are visualized as a heatmap (Figure 4). As shown by the HCL, there are major differences between the gene expression profiles of all the studied groups. Neither the chosen protocol, nor the macrophage subtype seem to be the main clustering factors. M1 and M2 macrophages polarized according to the Protocol B were clustered together with M0_C while M1 and M2 macrophages of the Protocol C were clustered with M1_A. Based on these results, the M1 and M2 macrophages polarized according to the Protocol A had the least similar transcriptomic profiles and therefore were not clustered together.

**Figure 5:**
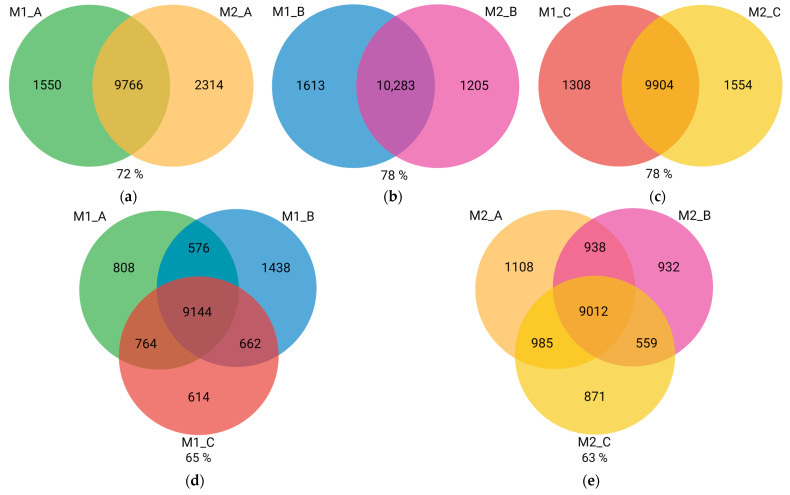
Venn diagrams of co-expression. Numbers of uniquely expressed and co-expressed genes are shown inside the circles. Percentages of the genes that are co-expressed out of all expressed genes are displayed below each diagram. (**a**) Protocol A; (**b**) Protocol B; (**c**) Protocol C; (**d**) M1 macrophages; (**e**) M2 macrophages. Created with BioRender.com; accessed on 3 February 2023.

Venn diagrams of co-expression supported the results from HCL analysis. Among all three polarization protocols, macrophage subtypes of the Protocol A shared the lowest number of co-expressed genes (72%) (Figure 5a–c). Moreover, the differences in transcriptome profiles were overall greater between Mφ of the same subtype but polarized according to different protocols than between different macrophage subtypes of the same protocol (on average 64% versus 76%) (Figure 5).

### 3.3. Differential Gene Expression Analysis

Based on the currently known gene signatures of M1 and M2 macrophages (reviewed in [2,5,12]) we composed gene sets which were further analyzed in the differential gene expression analysis. For both M1 and M2 macrophages, we composed three gene sets: regulators of macrophage polarization, cytokines and chemokines produced by Mφ, and receptors and enzymes (Table 1 and Table 2). In every gene set we display the comparisons between polarized macrophages and their respective M0 differentiation states, the M1 and M2 macrophages within one protocol, and M1 or M2 macrophages (depending on the gene set) between different protocols.

**Figure 6:**
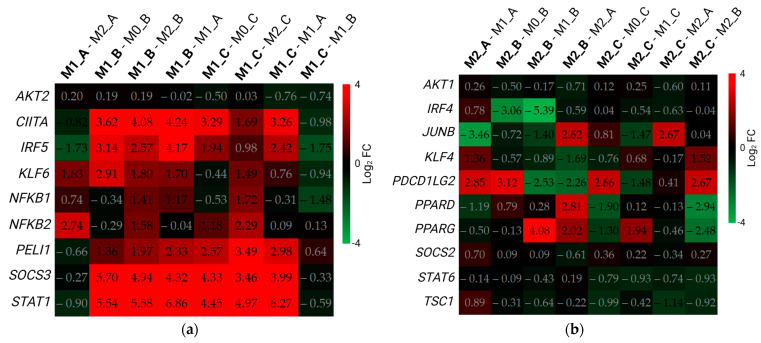
Differential expression of regulatory genes. Data in the figure are presented as log_2_ (FC). The threshold for differentially expressed genes was set as |log_2_ (FC)| ≥ 1 with an adjusted *p*-value of padj ≤ 0.05. DEGs are represented with digits in black font. Comparisons between different samples are displayed in the upper part of the figure along the x-axis. FC—fold change. DEGs—differentially expressed genes. (**a**) M1 gene set; (**b**) M2 gene set. Created with BioRender.com; accessed on 14 November 2022.

Firstly, we analyzed the M1 gene set of regulators of macrophage polarization (Figure 6a). The most over-expressed genes were *CIITA*, *SOCS3*, and *STAT1* in both the Protocols B and C. M1_B had the highest number of over-expressed genes in this set when compared with M0 macrophages, and M2_B and M1 macrophages from the other polarization protocols, although the differences between M1_B and M1_C were minimal.

Regarding the M2 set of regulatory genes (Figure 6b), many genes were not differentially expressed in any comparison (e.g., *AKT1*, *SOCS2*, *STAT6*, and *TSC1*). Surprisingly, only a few genes were over-expressed in M2 macrophages when comparing with M1 macrophages from their respective protocols (e.g., *KLF4* and *PDCD1LG2* in M2_A; *PPARG* in M2_B and M2_C). *PDCD1LG2* was over-expressed in M2 macrophages from Protocol B and Protocol C when compared with their respective M0 differentiation statuses. Some of the genes were even under-expressed in M2 macrophages when compared with M1 macrophages (e.g., *IRF4*, *JUNB*, and *PPARD*; *PDCD1LG2* in M2_B and M2_C).

**Figure 7:**
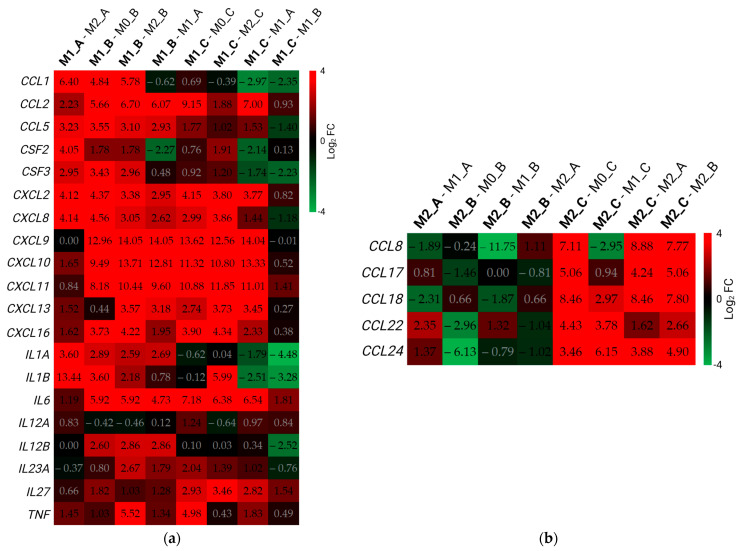
Differential expression of cytokine and chemokine genes. Data in the figure are presented as log_2_ (FC). The threshold for differentially expressed genes was set as |log_2_ (FC)| ≥ 1 with an adjusted *p*-value of padj ≤ 0.05. DEGs are represented with digits in black font. Comparisons between different samples are displayed in the upper part of the figure along the x-axis. FC—fold change. DEGs—differentially expressed genes. (**a**) M1 gene set; (**b**) M2 gene set. Created with BioRender.com; accessed on 14 November 2022.

In the cytokine and chemokine M1 gene set (Figure 7a), almost all genes were over-expressed in M1 macrophages compared with M0 and M2 macrophages. The highest expression was in M1 macrophages polarized according to Protocol B. On the other hand, most genes in M1_A were under-expressed when compared with M1_B and M1_C. Among the cytokine genes, the most differentially expressed genes (DEGs) in M1_B and M1_C were *CXCL9*, *CXCL10*, and *CXCL11*. In these macrophage subtypes, *IL6* had the highest log_2_ (FC) among the DEGs belonging to the cytokine group.

The M2 cytokine and chemokine set consists of five chemokine genes (Table 2). The highest number of DEGs in this gene set was in M1 macrophages polarized according to Protocol C (Figure 7b). *CCL18, CCL22,* and *CCL24* were over-expressed in M2_C when compared with M0_C, M1_C, and M2 macrophages from Protocols A and B. *CCL8* and *CCL17* were also over-expressed in M2_C in all comparisons with M2_C-M1_C. M1 macrophages had a higher expression of *CCL8* compared with M2_C, and *CCL17* was not differentially expressed in these samples. Besides *CCL22,* all chosen genes were under-expressed in M2_B macrophages when compared with M0_B and M1_B. Moreover, only two genes (*CCL22* and *CCL24*) were over-expressed in M2_A macrophages when compared with M1_A.

**Figure 8:**
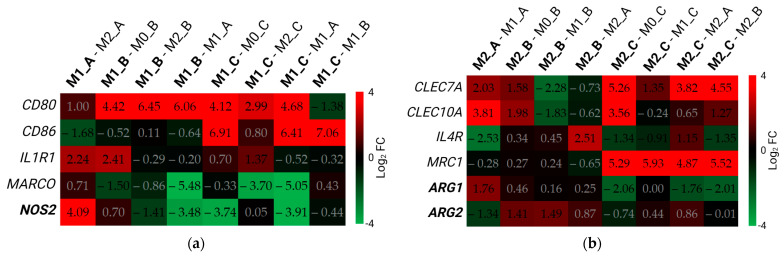
Differential expression of genes for receptors and enzymes. Data in the figure are presented as log_2_ (FC). The threshold for differentially expressed genes was set as |log_2_ (FC)| ≥ 1 with an adjusted *p*-value of padj ≤ 0.05. DEGs are represented with digits in black font. Comparisons between different samples are displayed in the upper part of the figure along the x-axis. Genes for enzymes are displayed in bold letters. FC—fold change. DEGs—differentially expressed genes. (**a**) M1 gene set; (**b**) M2 gene set. Created with BioRender.com; accessed on 14 November 2022.

The M1 gene set of genes coding receptors and enzymes (Figure 8a) consisted of four receptor genes (*CD80*, *CD86*, *IL1R1*, *MARCO*) and one gene for the inducible nitric oxide synthase (*NOS2*). Based on the comparisons between M1 macrophages and the respective M0 and M2 polarization states, we assume the expression of the *CD80* gene was up-regulated in M1 macrophages of all three protocols. Based on the comparisons shown in Figure 8a and gene quantification data (not shown), the expression of *CD80* was the highest in M1_B. *CD86* was over-expressed only in M1_C, while *NOS2* was over-expressed only in M1_A. Besides M1_A, *IL1R1* was over-expressed in M1_B and M1_C but not in all comparisons.

The M2 gene set (Figure 8b) consisted of four gene-coding receptors (*CLEC7A*, *CLEC10A*, *IL4R*, and *MRC1*) and two genes for arginase (*ARG1* and *ARG2*). Out of this gene set, *MRC1* and *CLEC7A* were over-expressed in M2_C compared with all other protocols and polarization states. *CLEC10A* was also over-expressed in M2_C when compared with M0_C and M2_B but not compared with M1_C and M2_A. While M2_A cells over-expressed *ARG1*, M2_B macrophages over-expressed *ARG2* compared with other cells from their respective protocols.

### 3.4. GO Enrichment Analysis

We next performed the GO (gene ontology) enrichment analysis to identify functional enrichment in genes that were differentially expressed in our samples. Firstly, we determined GO enrichment in genes significantly expressed in M1 macrophages from Protocols B and C versus their respective M0 differentiation statuses (Table 3 and Table 4). We also included the comparisons between different protocols. Since M1_ B seemed to differentially express the highest number of pro-inflammatory genes, in this analysis, we focused mainly on the macrophages from the Protocol B. Therefore, we do not include comparisons we considered less relevant (e.g., M1_A versus M1_C, etc.)

Compared with M0 macrophages, M1_B and M1_C up-regulated genes involved in inflammatory immune response, such as defense response to another organism (GO:0098542), response to bacterium (GO:0009217), response to molecules of bacterial origin (GO:0002237), or response to interferon gamma (GO:0034341) (Table 3 and Table 4).

We also identified functional enrichment in genes that were differentially expressed in M1_B when compared with M1 macrophages polarized according to Protocols A (Table 5) and C (Table 6). 

Next, we compared functional enrichment in M2 macrophages polarized according to the three chosen protocols. As a first step, we compared M2_B and M2_C macrophages with their respective M0 differentiation statuses. Based on the primordial comparisons, M2_B seemed to be involved in more M2-like biological processes, therefore, we focused on Protocol B. Similar to the GO enrichment analysis of M1 macrophages, we do not include comparisons we considered less relevant (e.g., M2_A versus M2_C, etc.).

Analysis of the differentially expressed genes between M2_B and M0_B revealed functional enrichment for blood vessel morphogenesis (GO:0048514), phagocytosis (GO:0006909), angiogenesis (GO:0001525), extracellular matrix organization (GO:0030198), and extracellular structure organization (GO:0043062) among the top 10 significantly enriched biological processes (Table 7).

When comparing M2 and M0 macrophages polarized according to Protocol C, we identified functional enrichment for biological processes mainly involved in regulation of protein translation and localization (e.g., GO:0006614, GO:0006613, GO:0045047, GO:0072599, and GO:0070972) (Table 8).

In the next step we compared M2 macrophages polarized according to the chosen protocols. DEGs in M2_B showed functional enrichment in biological processes involved in blood vessel morphogenesis (GO:0048514) and angiogenesis (GO:0001525) when compared with M2_A (Table 9) and M2_C (Table 10). When comparing M2_B and M2_A, we also identified functional enrichment for the response to molecules of bacterial origin (GO:0002237) and positive regulation of the response to an external stimulus (GO:0032103). Biological processes of extracellular matrix organization (GO:0030198) and extracellular structure organization (GO:0043062) were enriched in genes differentially expressed in M2_B versus M2_C.

## 4. Discussion

Macrophages are important cells of innate immunity with many functions regarding defense against pathogens, inflammatory processes, and anti-tumor activities. Moreover, they play a key part in tissue regeneration, wound healing, and clearance of old cells and debris [29,30]. These processes are carried out by different subtypes of Mφ polarized in response to stimuli present in their micro-environment [5]. Immortalized monocytic cell lines, such as THP-1, are often used to study the differentiation and function of Mφ [31]. In order to achieve results from these studies that would accurately predict the function and responses of in vivo tissue Mφ, different polarization protocols have been proposed and are regularly optimized. Still to this date, there is not a consensus in the scientific community regarding the most suitable protocol to achieve Mφ which would resemble the macrophages in a living organism the best [13,17,21].

Nowadays, gene and protein expression differences are considered to be the clearest and simplest way to distinguish the polarization state of macrophages [5]. Many authors analyze the gene expression or the whole transcriptome to characterize the polarization status of Mφ under different experimental conditions or in various diseases [32,33,34,35]. Some reviews try to summarize these data and present gene expression panels for M1 and M2 macrophages [5].

Therefore, in this study we carried out an RNA-seq transcriptome analysis of M1 and M2 macrophages polarized according to three different protocols. Although all three protocols were supposed to generate inflammatory M1 and anti-inflammatory M2 macrophages from THP-1 monocytes, we found great differences between macrophages generated by these protocols at the gene expression level, and also at the morphological level. Several authors have reported that polarized M1 macrophages have an elongated spindle-like morphology, while M2 macrophages are more roundly shaped [36,37,38,39,40]. Some of the cells in M1_A and M1_B samples had an elongated morphology, while M1_C macrophages had a round or rugged shape even though LPS was reported to stimulate the formation of spindle-like morphology in M1 macrophages [39]. M2 macrophages from all protocols were rounded, but occasionally we observed spindle-like cells in the M2_B sample. It should be noted that the data about macrophage morphology is contradictory since there are also studies reporting round M1 cells and spindle-like M2 cells [41,42,43]. Most of the M1_B macrophages had several projections, which were either smooth or ramified. Some papers have already described similar morphology of M1 macrophages polarized from monocytic cell lines [44,45] or peripheral blood monocytes [46,47]. Projections of the cell surface of macrophages allow them to move and to survey the extracellular milieu. Macrophage projections, e.g., filopodia, lamellipodia, and dorsal ruffles are essential for the migration and chemotaxis of immune cells. Moreover, they function in pathogen detection via toll-like receptors [48]. Later studies also proved that macrophage filopodia can pull pathogens towards the cell body and are crucial in the initiation of phagocytosis [49,50]. We can assume that due to their numerous cell projections, M1_B macrophages would be able to readily migrate towards pathogens and detect them. However, this hypothesis should be confirmed with further testing. It should be also noted that in our morphological analysis Protocol C differed from the other two chosen protocols also in regard to cell numbers. In Protocol C, we observed far less cells despite seeding the same number of THP-1 monocytes in all protocols prior to adding polarization stimuli. Since we did not observe any dead cells, we assume that the reduced number of cells was not due to the effect of the polarization stimuli on cell viability. On the other hand, reduced adherence of Mφ could offer a possible explanation for low cell numbers in Protocol C. A study by Lund et al. (2016) proved that both the concentration of PMA and the length of the post-stimulation PMA-deprivation period have a major effect on the adherence of THP-1 cells. According to this study, lower concentrations of PMA and longer rest periods reduce the adherence of stimulated THP-1 cells with only around 50% of cells remaining attached to the dish after exposure to PMA with a concentration of 8 ng/mL followed by a 48 h PMA-deprivation period [23]. Therefore, Mφ polarized according to Protocol C (PMA concentration c = 5 ng/mL; rest period 72 h) could have a reduced adherence which would result in unwanted aspiration of cells during the change in the growth medium and/or removal of PMA.

Major differences between the polarization protocols were also confirmed and further studied via the transcriptome analysis. Firstly, when comparing transcriptomic profiles using HCL, we found out that neither the macrophage subtype nor the polarization protocol seemed to be the main clustering factor. Despite that, M1 and M2 macrophages from Protocol B were clustered together (along with M0_C) and separated from the other samples. Moreover, M1_C and M2_C were also clustered closely together. Therefore, the differences between polarization protocols seemed to be greater than differences between M1 and M2 macrophages, especially in Protocol B. Since M1 and M2 macrophages should in many ways have opposite functions, they tend to separate in HCL [35]. Therefore, we expected that the macrophage subtype would be a better clustering factor than the polarization protocol. However, this was not proven by the HCL. According to both the HCL and Venn diagrams of co-expression, the greatest differences between M1 and M2 macrophages were in Protocol A. Experiments using THP-1 and monocytic cell line HL-60 have revealed that PMA induces a greater degree of differentiation than Vit D_3_ [51]. HL-60 cells stimulated with Vit D_3_ were observed to have a monocytic phenotype [52] while phorbol esters (e.g., PMA) induce differentiation into macrophage-like cells [53]. The degree of differentiation may induce greater differences in gene expression profiles than polarization into M1 or M2 subtypes; therefore, differences between macrophage polarization subtypes in Protocol A would be greater than between the respective subtypes in Protocols B and C.

Our analysis of DEGs focused mainly on three gene sets: regulators of macrophage polarization, cytokines and chemokines produced by Mφ, and macrophage receptors and enzymes. Regarding the regulators of M1 macrophage polarization, we observed the highest number of genes with significant over-expression in M1_B when compared with M0_B and M1 macrophages polarized according to other polarization protocols. The only gene that was not over-expressed was *AKT2,* which is a part of the PI3K/Akt/mTORC1 signaling [54]. Due to the activity of the PI3K/Akt pathway, differentiated macrophages are resistant to apoptotic stimuli. Moreover, differential expression of *AKT1* and *AKT2* homologues regulates immune response, in which the *AKT2* homologue stimulates inflammation. Many studies have confirmed that *AKT2* ^(−/−)^ deletion promotes the anti-inflammatory M2 phenotype while *AKT1* ^(−/−)^ deletion skews cells towards the M1 phenotype [55]. There were no significant differences between the expression of *AKT2* in our samples. However, it is important to note that studies proposing the role of *AKT2* in M1 macrophages used genetically modified animals and cells with deletion of *AKT2* [55,56]. Therefore, lack of publications regarding the expression of *AKT2* in M1 and M2 macrophages without genetic modification makes it difficult to establish whether *AKT2* is differentially expressed between M1 and M2 macrophages. Overall, M1_A and M1_C showed lower levels of the regulatory genes’ expression compared with M1_B. 

When analyzing the M1 cytokine and chemokine gene set we obtained similar results. The highest number of over-expressed genes was in M1_B which may suggest the highest production of inflammatory cytokines and chemokines in M1_B cells. However, a study by Raza et al. proved that some of the chemokine genes (including *CXCL9*, *CXCL10*, and *CXCL11*) reach their highest expression level between 8–24 h post stimulation by LPS [57]. Therefore, RNA from M1_B cells (stimulated with cytokines for 18 h) was isolated right in the window with the highest expression of the aforementioned chemokines. The only gene that was not differentially expressed in M1_B when compared with M1 macrophages from other polarization protocols and M0_B was *IL12A*. This gene codes inflammatory cytokine which drives macrophages toward the M1 phenotype. Moreover, M1 macrophages are the main source of IL-12 [58,59]. Our results show that there was no significant difference between the levels of expression of *IL12A* in Mφ subtypes and different protocols (except when comparing M1_C and M0_C) even though expression of this gene is heavily associated with M1 macrophages [59].

The analysis of the M1 receptor and enzyme set showed that only the *CD80* gene was over-expressed in M1 macrophages polarized by any of the three tested protocols when compared with M2 macrophages, with the highest level of expression in M1_B. CD80 is a membrane receptor on the surface of antigen-presenting cells including macrophages, and it also serves as a ligand to CD28 on T-lymphocytes and promotes T-cell response [60]. CD80 is considered one of the key markers of M1 macrophages with the power to discriminate between M1 and M2 polarization states [61,62]. Another ligand to CD28 is CD86, which was over-expressed only in M1_C. CD86 is also considered an M1 polarization marker, but its role in T-cell activation remains elusive. Although some studies have shown that CD86 is the dominant ligand for regulation of immune response and proliferation of regulatory T-cells (Treg) [63,64], other authors report the role of CD86^+^ macrophages in inflammatory response [65], and favorable prognosis in colorectal cancer and hepatocellular carcinoma possibly due to a higher degree of M1 polarization and the subsequent anti-tumor activities [66,67]. It was also reported that the presence of CD86^+^ macrophages correlated with the severity of tubulointerstitial inflammation in human glomerulonephritis [68]. However, it should be noted that M1_B macrophages maybe did not express *CD86* due to a short exposure time. A study by Parise et al. showed that most chemical sensitizers induced the transcription of *CD86* in THP-1 cells only after a 48 h of exposure to the chosen sensitizer [69]. Therefore, Mφ polarized according to Protocol B could not up-regulate the transcription of *CD86*, since they were exposed to cytokine stimuli for only 18 h. The gene for the IL-1 receptor was over-expressed in M1_A and M1_C macrophages when compared with M2 macrophages from their respective polarization protocols while *IL1R1* was over-expressed in M1_B when compared with M0_B but not when compared with M2_B. According to a study by Baxter et al., the rest period between the M0 state and the polarization of M2 macrophages is necessary for the down-regulation of M1 gene expression [21]. A relatively high concentration of PMA combined with the lack of a rest period in Protocol B may have caused the expression of *IL1R1* to not be down-regulated during the polarization of M2_B; therefore, it was not differentially expressed when comparing M1 and M2 macrophages from Protocol B. Moreover, *IL1R1* was not over-expressed in M1_C when compared with M0_C probably because PMA on its own can be considered an M1 polarization stimulus [70] and can up-regulate the transcription of M1-associated genes [21]. Surprisingly, the *MARCO* gene was not differentially expressed in many of the comparisons. In addition, the expression of *MARCO* was higher in M2_C when compared with M1_C and in M0_B when compared with M1_B. MARCO is a member of the class A scavenger receptor (SR-A) family and is mainly expressed on macrophages [71]. This receptor is considered a marker of M1 macrophages [72] mainly because it functions as a major phagocytic receptor mediating the binding and uptake of bacteria [73] and viruses [74]. Despite these functions, recent studies have found a positive correlation between the expression of *MARCO* and production of anti-inflammatory cytokine IL-37. Similarly, MARCO^+^ macrophages enhanced Treg cell proliferation and IL-10 production [75]. Additionally, Chen et al. reported that MARCO^+^ macrophages drive tumor progression in glioblastomas and proposed that MARCO could be used as a mesenchymal pro-tumor marker [76]. The only enzyme-coding gene in this M1 panel is the *NOS2* gene, which codes the enzyme nitric oxide synthase 2. This enzyme, along with the arginase 1 and arginase 2 enzymes (coded by *ARG1* and *ARG2* genes), represent key molecules in macrophage polarization and are closely linked to the functional phenotype of Mφ [2]. Depending on the stimuli present in their surrounding micro-environment, Mφ, in the process of polarization, alter their arginine metabolism by which they “decide” to execute the “inhibit” or “heal” function [12]. In the presence of inflammatory stimuli, Mφ can metabolize arginine into nitric oxide (NO) and citrulline by the means of the up-regulated NOS enzyme. NO production is an important effector for the anti-microbial activity of M1 macrophages [2,77]. To execute the “heal” function, Mφ actively metabolize arginine by arginase into ornithine and urea. Macrophage production of ornithine is essential for many repair processes because it serves as a precursor of the polyamines required for cell proliferation. Ornithine also serves as a precursor of collagen which is important for the construction of the extracellular matrix [78]. Despite the crucial role of NOS2 in M1 macrophages and the dichotomy of NOS/ARG expression being considered the hallmark of macrophage polarization, we have found that *NOS2* was over-expressed only in M1_A. The overall expression of *NOS2* gene in all samples was very low based on the gene expression quantification data (FPKM < 2.2). However, several studies have already shown that this heal/inhibit dichotomy based on the changes in arginase metabolism does not apply equally to human and mouse Mφ [79]. While mouse Mφ express NOS and produce high amounts of NO as a response to inflammatory stimuli, human macrophages make little to no NO due to the high level of methylation around the *NOS2* transcription start site [80]. Therefore, the use of the expression and activity of NOS/ARG for the determination of the macrophage function and subtype should be reassessed. It should be noted that differences between Mφ generated by different protocols could arise due to different times of exposure to polarization stimuli. Firstly, it has been proven that exposure time can significantly alter the effect of the stimuli, including promoting or inhibiting the transcription of genes [81]. Moreover, the inflammatory response includes early response genes, which are transcribed almost immediately after the exposure to the stimulus, and late response genes, which are transcribed later on [82]. An extensive study of murine macrophages by Raza et al. divided the transcribed genes into three clusters: early, mid, and late responses [57]. Since each protocol implemented different exposure times and we performed the RNA isolation immediately after removing the polarization stimuli, our data represent the transcriptome profiles of macrophages in different time points. Therefore, differences between gene expression levels of macrophages from the three chosen protocols may not mean that these cells express the analyzed genes in a different way. It may simply mean that the gene expression was analyzed at a different time for each protocol and therefore cannot be compared.

The differential gene expression of M2 macrophages was also analyzed. Most of the genes in both the regulatory gene set and the panel of genes for receptors and enzymes were not differentially expressed in M2 macrophages when comparing with other cells and protocols, which suggests that the expression of these genes was not up-regulated in the M2 macrophages. The causes differ between the chosen polarization protocols. Protocol A uses Vit D_3_ for the polarization of M2 macrophages [22]. Even though this compound is usually considered an M2 macrophage polarization stimulus [83,84,85,86], there is evidence regarding its effect on the up-regulation of the expression of inflammatory cytokines and antimicrobial peptides [36], increase in the M1/M2 macrophage ratio [87], or enhancement of bactericidal activity and superoxide production in macrophages [88], which are all associated with the M1 phenotype. Moreover, Neme et al. reported that gene ontology assessment of the Vit D_3_-stimulated THP-1 monocytes revealed anti-microbial response as their top-ranking early physiological function [89]. Therefore, the aforementioned studies along with our results suggest that the role of Vit D_3_ as an M2 macrophage stimulator should be reassessed. The effect of the stimulation time should also be taken into consideration. A recent study by Unuvar Purcu et al. revealed the effect of the stimulation time on the expression of macrophage markers [90]. According to their study, M2 macrophages had the highest level of *MRC1* and *CCL22* expression after 48–72 h of stimulation. Since macrophages in Protocol B were stimulated with IL-4 and IL-13 for 18 h, this time was probably not sufficient to up-regulate the expression of *MRC1* and *CCL22,* unlike in Protocol C where the M0 cells were stimulated with IL-4 for 48 h. The relatively high concentration of PMA used in Protocol B (61.3 ng/mL) could be another reason for the failed expression of M2-associated genes. A study by Chanput et al. suggests that initial priming with a high concentration of PMA (100 ng/mL) compromises polarization of M2 macrophages [91]. Baxter et al. also confirmed that priming of THP-1 cells with concentrations of PMA above 50 ng/mL inhibited transcription of M2-associated genes [21]. Based on these studies, we also incorporated Protocol C in our experiment, which is suggested for the polarization of M2 macrophages by Baxter et al. [21]. It should be noted that their study analyzed the expression of only a few chosen M2-related genes (*MRC1*, *CD200R*, *CCL17*, *ALOX15*, and *TGM2*). These genes were also over-expressed in M2_C macrophages in our study (see Figure 6b and Figure 7b; data for *CD200R*, *ALOX15,* and *TGM2* are not shown). However, our transcriptome analysis showed that M2_C cells failed to up-regulate other genes which are associated with the M2 phenotype. Only the genes for chemokines along with *MRC1* and *CLEC7A* were significantly over-expressed when comparing with other macrophage subtypes and polarization protocols. Therefore, further optimization of the M2-polarization protocol is needed.

Results from the differential gene expression analysis were supported by the GO enrichment analysis. DEGs of M1_B (when compared with M0_B) were functionally enriched in biological processes which are generally associated with the function of M1 macrophages [2], e.g., defense response to another organism (GO:0098542), response to bacterium (GO:0009217), adaptive immune response (GO:0002250), response to a virus (GO:0009615), or defense response to a virus (GO:0051607). We also identified functional enrichment for M1-associated processes in genes that were differentially expressed in M1_C when comparing with M0_C, e.g., defense response to another organism (GO:0098542), response to bacterium (GO:0009217), regulation of immune effector process (GO:0002697), and activation of immune response (GO:0002253). However, comparisons between M1_B, M1_A, and M1_C revealed that the M1-associated processes mentioned earlier (see Table 5 and Table 6) were over-represented in M1 macrophages polarized according to Protocol B. These results suggest that M1_B could have the strongest antimicrobial, antiviral, and overall defense response out of all tested M1 macrophages.

According to the differential gene expression analysis, neither of the protocols could be considered a reliable model for the polarization of M2 macrophages. Even though M2_C cells expressed some of the genes that are associated with the M2 phenotype (e.g., genes for chemokines: *MRC1* and *CLEC7A*) [2,5], functionally (see Table 8) they could not be considered as M2 macrophages. On the other hand, M2_B did not differentially express the genes for M2 regulatory factors, chemokines, or receptors and enzymes, but according to the GO enrichment analysis they could perform some of the biological processes which are traditionally associated with the M2 phenotype. It has been proven that M2 macrophages promote angiogenesis [92,93], remodeling of the extracellular matrix, phagocytosis of old and dead cells, and coordination of tissue regeneration (reviewed in [12,94,95]). We identified some of these processes among the GO terms significantly enriched in DEGs of M2_B macrophages: e.g., phagocytosis (GO:0006909), collagen metabolic process (GO:0032963), angiogenesis (GO:0001525), or extracellular matrix organization (GO:0030198). As previously mentioned, Baxter et al. proved that priming THP-1 monocytes with relatively high concentrations of PMA could block the expression of M2-associated genes [21]. However, the reasons why the M2 macrophages from Protocol B lack the established markers for M2 polarization but show enrichment for biological processes of the M2 macrophages should be further investigated and supported by experiments.

Our study provided new remarkable information regarding the reliability of commonly used macrophage polarization protocols. Despite that, we acknowledge the limitations of our study. First, transcriptomic data are not supported by RT-PCR or proteomic analysis; therefore, conclusions from this study need to be supported by further experiments. Moreover, this research is primarily a hypothesis-generating study, rather than a hypothesis-testing study.

## 5. Conclusions

In conclusion, our study provided a comparison of several widely used macrophage polarization protocols at the transcriptome level. Based on the results, we assume that M1 macrophages polarized according to Protocol B represent a reliable model of inflammatory macrophages with high expression of genes for regulatory factors, cytokines, and chemokines. M1_B also showed functional enrichment in processes typically associated with the M1 phenotype. These cells did not over-express some of the genes for M1-associated receptors and enzymes. However, traditional markers should be reassessed, mainly regarding the differences in their expression between various animal species. On the other hand, according to our results, none of the tested protocols is suitable for the polarization of M2 macrophages. These protocols are used in a great number of studies regarding the function of M2 macrophages and their role in the pathophysiology of numerous diseases. Therefore, it is necessary to test new protocols or optimize the currently used ones to achieve a way to polarize reliable anti-inflammatory macrophages that could be used in further research.

## Figures and Tables

**Table 1:** M1 gene sets for differential gene expression analysis.

Gene Set	Gene Symbol	Gene Name
Regulators of macrophage polarization	*AKT2*	AKT Serine/Threonine Kinase 2
	*CIITA*	Class II Major Histocompatibility Complex Transactivator
	*IRF5*	Interferon Regulatory Factor 5
	*KLF6*	KLF Transcription Factor 6
	*NFKB1*	Nuclear Factor Kappa B Subunit 1
	*NFKB2*	Nuclear Factor Kappa B Subunit 2
	*PELI1*	Pellino E3 Ubiquitin Protein Ligase 1
	*SOCS3*	Suppressor of Cytokine Signaling 3
	*STAT1*	Signal Transducer and Activator of Transcription 1
Cytokines and chemokines	*CCL1*	C-C Motif Chemokine Ligand 1
	*CCL2*	C-C Motif Chemokine Ligand 2
	*CCL5*	C-C Motif Chemokine Ligand 5
	*CSF2*	Colony Stimulating Factor 2
	*CSF3*	Colony Stimulating Factor 3
	*CXCL2*	C-X-C Motif Chemokine Ligand 2
	*CXCL8*	C-X-C Motif Chemokine Ligand 8
	*CXCL9*	C-X-C Motif Chemokine Ligand 9
	*CXCL10*	C-X-C Motif Chemokine Ligand 10
	*CXCL11*	C-X-C Motif Chemokine Ligand 11
	*CXCL13*	C-X-C Motif Chemokine Ligand 13
	*CXCL16*	C-X-C Motif Chemokine Ligand 16
	*IL1A*	Interleukin 1 Alpha
	*IL1B*	Interleukin 1 Beta
	*IL6*	Interleukin 6
	*IL12A*	Interleukin 12A
	*IL12B*	Interleukin 12B
	*IL23A*	Interleukin 23 Subunit Alpha
	*IL27*	Interleukin 27
	*TNF*	Tumor Necrosis Factor
Receptors and enzymes	*CD80*	CD80 Molecule
	*CD86*	CD86 Molecule
	*IL1R1*	Interleukin 1 Receptor Type 1
	*MARCO*	Macrophage Receptor With Collagenous Structure
	*NOS2*	Nitric Oxide Synthase 2

**Table 2:** M2 gene sets for differential gene expression analysis.

Gene Set	Gene Symbol	Gene Name
Regulators of macrophage polarization	*AKT1*	AKT Serine/Threonine Kinase 1
	*IRF4*	Interferon Regulatory Factor 4
	*JUNB*	JunB Proto-Oncogene, AP-1 Transcription Factor Subunit
	*KLF4*	KLF Transcription Factor 4
	*PDCD1LG2*	Programmed Cell Death 1 Ligand 2
	*PPARD*	Peroxisome Proliferator Activated Receptor Delta
	*PPARG*	Peroxisome Proliferator Activated Receptor Gamma
	*SOCS2*	Suppressor of Cytokine Signaling 2
	*STAT6*	Signal Transducer and Activator of Transcription 6
	*TSC1*	TSC Complex Subunit 1
Cytokines and chemokines	*CCL8*	C-C Motif Chemokine Ligand 8
	*CCL17*	C-C Motif Chemokine Ligand 17
	*CCL18*	C-C Motif Chemokine Ligand 18
	*CCL22*	C-C Motif Chemokine Ligand 22
	*CCL24*	C-C Motif Chemokine Ligand 24
Receptors and enzymes	*CLEC7A*	C-Type Lectin Domain Containing 7A
	*CLEC10A*	C-Type Lectin Domain Containing 10A
	*IL4R*	Interleukin 4 Receptor
	*MRC1*	Mannose Receptor C-Type 1
	*ARG1*	Arginase 1
	*ARG2*	Arginase 2

**Table 3:** Top 10 biological processes significantly (padj ≤ 0.01) enriched in genes differentially expressed in M1_B versus M0_B macrophages.

GO ID	Biological Process	GeneRatio
GO:0034341	Response to interferon gamma	84/2236
GO:0098542	Defense response to another organism	125/2236
GO:0071346	Cellular response to interferon gamma	71/2236
GO:0002250	Adaptive immune response	109/2236
GO:0060333	Interferon-gamma-mediated signaling pathway	45/2236
GO:0009617	Response to bacterium	133/2236
GO:0051607	Defense response to virus	79/2236
GO:0009615	Response to virus	99/2236
GO:0002237	Response to molecule of bacterial origin	104/2236
GO:0060337	Response to type I interferon	43/2236

**Table 4:** Top 10 biological processes significantly (padj ≤ 0.01) enriched in genes differentially expressed in M1_C versus M0_C macrophages.

GO ID	Biological Process	GeneRatio
GO:0009617	Response to bacterium	142/2341
GO:0098542	Defense response to another organism	126/2341
GO:0034341	Response to interferon gamma	73/2341
GO:0036230	Granulocyte activation	151/2341
GO:0042119	Neutrophil activation	148/2341
GO:0002697	Regulation of immune effector process	106/2341
GO:0002253	Activation of immune response	150/2341
GO:0002237	Response to molecule of bacterial origin	103/2341
GO:0043312	Neutrophil degranulation	143/2341
GO:0043299	Leukocyte degranulation	152/2341

**Table 5:** Top 10 biological processes significantly (padj ≤ 0.01) enriched in genes differentially expressed in M1_B versus M1_A macrophages.

GO ID	Biological Process	GeneRatio
GO:0034341	Response to interferon gamma	80/2561
GO:0060333	Interferon-gamma-mediated signaling pathway	49/2561
GO:0098542	Defense response to another organism	128/2561
GO:0051607	Defense response to virus	84/2561
GO:0009615	Response to virus	106/2561
GO:0002253	Activation of immune response	158/2561
GO:0002250	Adaptive immune response	110/2561
GO:0071346	Cellular response to interferon gamma	67/2561
GO:0009617	Response to bacterium	135/2561
GO:0002237	Response to molecule of bacterial origin	108/2561

**Table 6:** Top 10 biological processes significantly (padj ≤ 0.01) enriched in genes differentially expressed in M1_B versus M1_C macrophages.

GO ID	Biological Process	GeneRatio
GO:0051607	Defense response to virus	100/3377
GO:0009615	Response to virus	126/3377
GO:0001819	Positive regulation of cytokine production	152/3377
GO:0034341	Response to interferon gamma	88/3377
GO:0060333	Interferon-gamma-mediated signaling pathway	51/3377
GO:0002237	Response to molecule of bacterial origin	129/3377
GO:0007249	I-kappaB kinase/NF-kappaB signaling	110/3377
GO:0098542	Defense response to another organism	146/3377
GO:0009617	Response to bacterium	182/3377
GO:0045860	Positive regulation of protein kinase activity	181/3377

**Table 7:** Top 10 biological processes significantly (padj ≤ 0.01) enriched in genes differentially expressed in M2_B versus M0_B macrophages.

GO ID	Biological Process	GeneRatio
GO:0030198	Extracellular matrix organization	78/1552
GO:0043062	Extracellular structure organization	83/1552
GO:0048514	Blood vessel morphogenesis	101/1552
GO:0030574	Collagen catabolic process	24/1552
GO:0050900	Leukocyte migration	74/1552
GO:0032963	Collagen metabolic process	33/1552
GO:0006909	Phagocytosis	52/1552
GO:0001525	Angiogenesis	86/1552
GO:0007264	Small GTPase mediated signal transduction	89/1552
GO:0043299	Leukocyte degranulation	94/1552

**Table 8:** Top 10 biological processes significantly (padj ≤ 0.01) enriched in genes differentially expressed in M2_C versus M0_C macrophages.

GO ID	Biological Process	GeneRatio
GO:0006614	SRP-dependent cotranslational protein targeting to membrane	79/2714
GO:0006613	Cotranslational protein targeting to membrane	79/2714
GO:0045047	Protein targeting to ER ^1^	81/2714
GO:0072599	Establishment of protein localization to ER	81/2714
GO:0070972	Protein localization to ER	84/2714
GO:0000184	Nuclear-transcribed mRNA catabolic process, nonsense-mediated decay	78/2714
GO:0006612	Protein targeting to membrane	89/2714
GO:0006119	Oxidative phosphorylation	73/2714
GO:0019083	Viral transcription	89/2714
GO:0019080	Viral gene expression	93/2714

^1^ ER—endoplasmic reticulum.

**Table 9:** Top 10 biological processes significantly (padj ≤ 0.01) enriched in genes differentially expressed in M2_B versus M2_A macrophages.

GO ID	Biological Process	GeneRatio
GO:0048514	Blood vessel morphogenesis	153/2298
GO:0040017	Positive regulation of locomotion	132/2298
GO:0050900	Leukocyte migration	110/2298
GO:2000147	Positive regulation of cell motility	123/2298
GO:0030335	Positive regulation of cell migration	120/2298
GO:0051272	Positive regulation of cellular component movement	124/2298
GO:0043410	Positive regulation of MAPK cascade	125/2298
GO:0002237	Response to molecule of bacterial origin	90/2298
GO:0001525	Angiogenesis	123/2298
GO:0032103	Positive regulation of response to external stimulus	74/2298

**Table 10:** Top 10 biological processes significantly (padj ≤ 0.01) enriched in genes differentially expressed in M2_B versus M2_C macrophages.

GO ID	Biological Process	GeneRatio
GO:0030198	Extracellular matrix organization	89/2254
GO:0033674	Positive regulation of kinase activity	132/2254
GO:0048514	Blood vessel morphogenesis	130/2254
GO:0043062	Extracellular structure organization	93/2254
GO:0045860	Positive regulation of protein kinase activity	121/2254
GO:0048667	Cell morphogenesis involved in neuron differentiation	122/2254
GO:0001525	Angiogenesis	108/2254
GO:0006820	Anion transport	111/2254
GO:0035051	Cardiocyte differentiation	41/2254
GO:0001764	Neuron migration	40/2254

## Data Availability

RNA-seq data are available in the FASTQ format at the Sequence Read Archive (SRA) under the accession PRJNA930713.
